# Microarray analysis provides new insights into the function of apolipoprotein O in HepG2 cell line

**DOI:** 10.1186/1476-511X-12-186

**Published:** 2013-12-17

**Authors:** Chen-Lu Wu, Shui-Ping Zhao, Bi-Lian Yu

**Affiliations:** 1 Department of Cardiology, the Second Xiangya Hospital of Central South University, Middle Ren-Min Road No.139, Changsha, Hunan, 410011, PR China

**Keywords:** Apolipoprotein O, Inflammation, Lipid metabolism, Fatty acids

## Abstract

**Background:**

Apolipoprotein O (apoO) is a new member of the apolipoprotein family. However, data on its physiological functions are limited and inconsistent. Using a microarray expression analysis, this study explored the function of apoO in liver cells.

**Methods:**

HepG2 cells were treated either with oleic acid or tumor necrosis factor-α for 24 h. mRNA and protein expression of apoO were assessed by quantitative real-time PCR (qRT-PCR) and Western blot respectively. An efficient lentiviral siRNA vector targeting the human apoO gene was designed and constructed. The gene expression profile of HepG2 human hepatocellular carcinoma cells transfected with the apoO silencing vector was investigated using a whole-genome oligonucleotide microarray. The expression levels of some altered genes were validated using qRT-PCR.

**Results:**

ApoO expression in HepG2 cells was dramatically affected by lipid and inflammatory stimuli. A total of 282 differentially expressed genes in apoO-silenced HepG2 cells were identified by microarray analysis. These genes included those participating in fatty acid metabolism, such as *ACSL4*, *RGS16*, *CROT* and *CYP4F11,* and genes participating in the inflammatory response, such as *NFKBIZ, TNFSF15, USP2, IL-17, CCL23, NOTCH2*, *APH-1B* and *N2N*. The gene Uncoupling protein 2 (*UCP2*), which is involved in both these metabolic pathways, demonstrated significant changes in mRNA level after transfection.

**Conclusions:**

It is likely that apoO participates in fatty acid metabolism and the inflammatory response in HepG2 cells, and UCP2 may act as a mediator between lipid metabolism and inflammation in apoO-silenced HepG2 cells.

## Background

Apolipoprotein O (apoO) is a novel apolipoprotein which was first discovered in 2006 [1], but to date, relatively little is known about its physiological functions. An *in vitro* study displayed that purified recombinant apoO facilitated cholesterol efflux from J774 mouse macrophage cells [1]. However, this effect has not been replicated *in vivo*[2]. Recently, we found that apoO levels were increased in acute coronary syndrome patients and positively associated with the inflammatory marker high-sensitive C-reactive protein (hsCRP), suggesting a potential role as an inflammatory predictor [3]. Therefore, apoO seems to be implicated in lipid metabolism and inflammation.

Here, the effects of lipid and inflammatory stimuli on apoO were investigated. A microarray assay was used to identify genes that are differentially expressed in apoO-silenced HepG2 cells. This study could enable the function of apoO in liver cells to be elucidated.

## Results

### Oleic acid increased the expression of apoO in HepG2 cells

**Figure 1:**
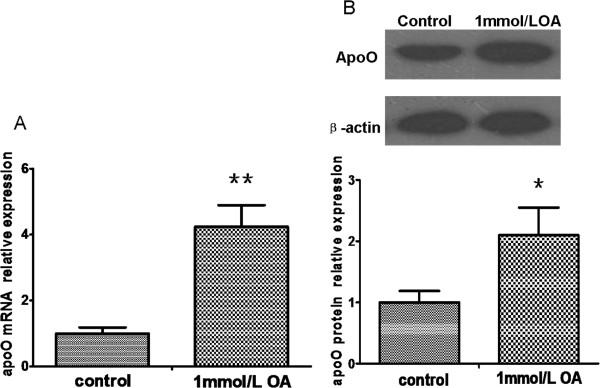
**ApoO mRNA and protein changes in HepG2 cells pretreated with 1 mmol/L OA for 24 h. A**. The relative levels of apoO mRNA were analyzed by qRT-PCR. Data shown are the mean ± S.E.M from experiments repeated in triplicate with three samples per treatment. **p < 0.01 vs. control. **B**. The relative levels of apoO protein were analyzed by Western blot analysis. β-actin served as a loading control. These experiments were performed three times, and the results of the densitometric analysis and one representative image are shown. * p < 0.05 vs. control.

We determined if a lipid stimulus would influence the expression of apoO in HepG2 cells. Figure 1 shows apoO mRNA and protein levels in cells treated with oleic acid (OA) compared with the levels in control cells. As expected, incubation of HepG2 cells with 1 mmol/L OA for 24 h induced a 4-fold increase in apoO mRNA (P < 0.01) and a 2-fold increase in apoO protein expression (P < 0.05).

### Tumor necrosis factor-α induced the expression of apoO in HepG2 cells

**Figure 2:**
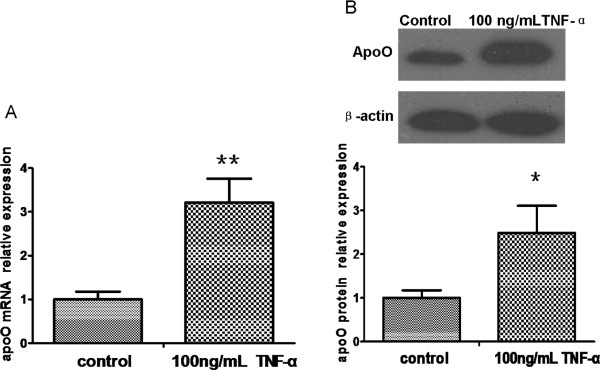
**Levels of ApoO mRNA and protein in HepG2 cells pretreated with 100 ng/mL TNF-α for 24 h. A**. The relative levels of apoO mRNA were analyzed by qRT-PCR. Data shown are the mean ± S.E.M from experiments repeated in triplicate with three samples per treatment. **p < 0.01 vs. control. **B**. The relative levels of apoO protein were analyzed by Western blot analysis. β-actin served as a loading control. These experiments were performed three times, and the results of the densitometric analysis and one representative image are shown. * p < 0.05 vs. control.

To explore the effect of inflammatory stimulus on the expression of apoO, we detected apoO mRNA and protein expression changes in HepG2 cells pretreated for 24 h with 100 ng/mL tumor necrosis factor-α (TNF-α). Quantitative real-time PCR (qRT-PCR) demonstrated that TNF-α treatment led to a 3-fold up-regulation of apoO mRNA expression (P < 0.01; Figure 2A). ApoO protein levels were approximately 2.5-fold higher after incubation (P < 0.05; Figure 2B).

### LV2 was identified as an efficient vector capable of silencing apoO

**Figure 3:**
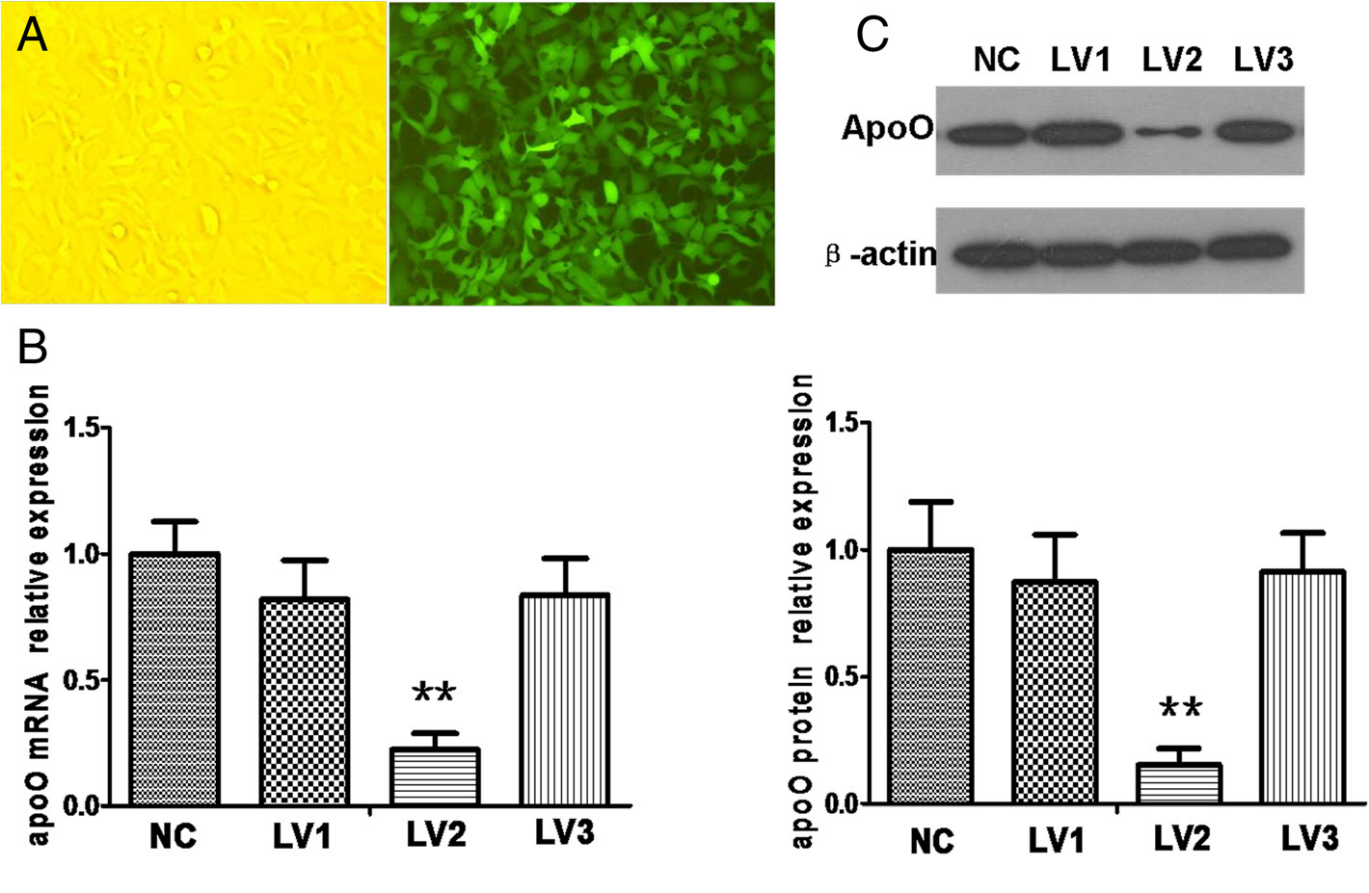
**Screening for an efficient lentiviral vector capable of silencing apoO expression. A**. HeLa cells observed by fluorescence microscopy 3 days after infection (magnification, ×200). **B**. The relative levels of apoO mRNA transcripts were analyzed by qRT-PCR. Data shown are means ± S.E.M from three independent experiments. **p < 0.001 vs. negative controls (NC). **C**. Effects of apoO silencing were measured using Western blot. NC: cells infected with negative control RNAi; LV1: cells infected with apoO-specific RNAi-1; LV2: cells infected with apoO-specific RNAi-2; LV3: cells infected with apoO-specific RNAi-3. β-actin served as a loading control. These experiments were performed three times, and the results of the densitometric analysis and one representative image are shown. **p < 0.001 vs. negative control (NC).

Using GenBank information for the human apoO gene, three interfering sequences and a negative control sequence were designed and designated as LV1, LV2, LV3 and NC. PCR identification and DNA sequencing demonstrated the correct insertion of the oligonucleotides into the vectors. qRT-PCR and Western blot analysis confirmed that LV2 could significantly inhibit apoO expression in HeLa cells when the multiplicity of infection (MOI) =10 (P < 0.001; Figure 3), whereas LV1 and LV3 had no apparent effect (P > 0.05).

### The LV2 lentiviral vector could dramatically suppress apoO expression in HepG2 cells

**Figure 4:**
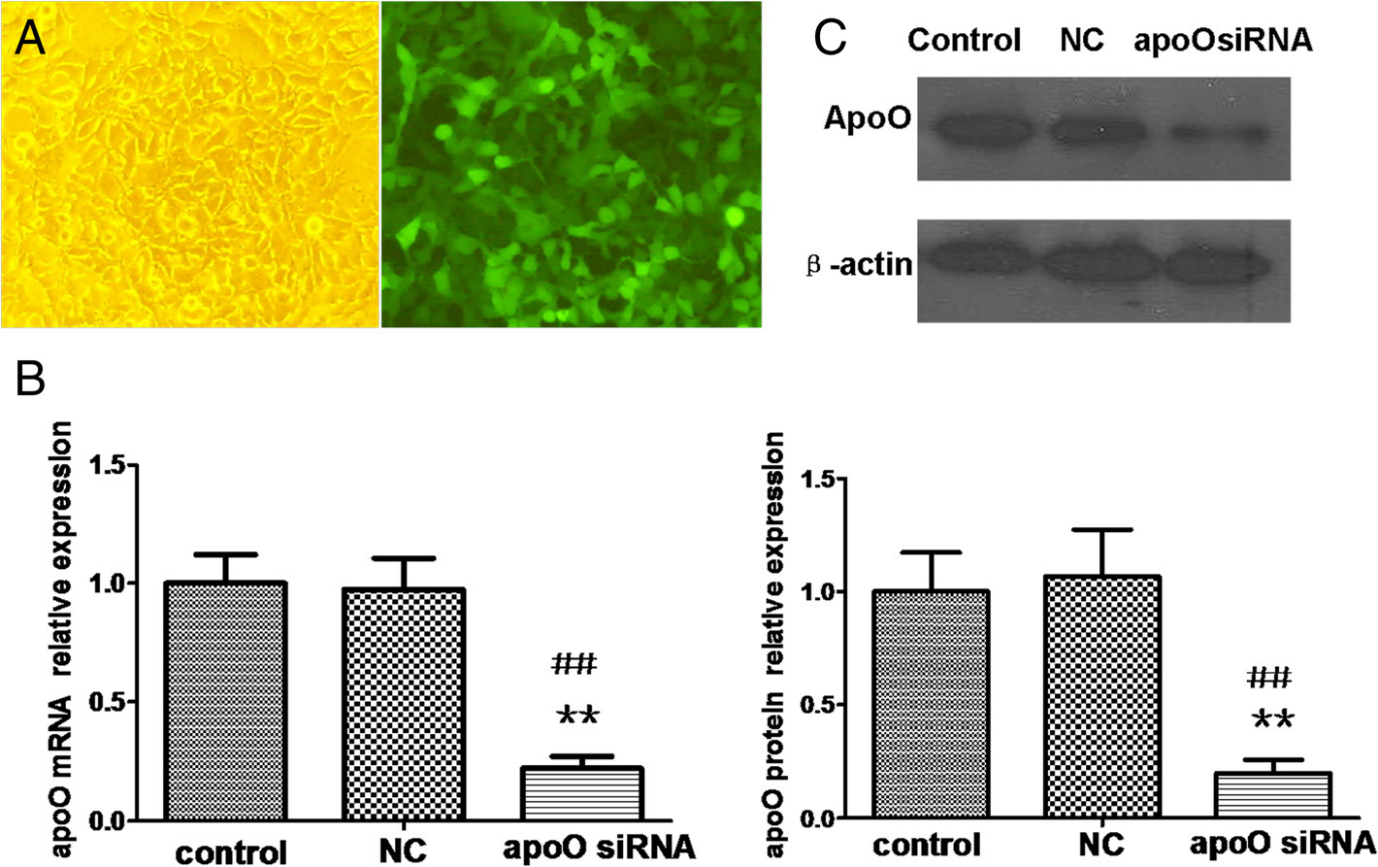
**Down-regulation of apoO expression in HepG2 cells using lentivirus-mediated RNAi. A**. HepG2 cells were observed by fluorescence microscopy 3 days after infection (magnification, ×200). **B**. The relative levels of apoO mRNA were analyzed by qRT-PCR. Data shown are the means ± S.E.M of experiments repeated three times with triplicate samples per treatment. **p < 0.01 vs. NC; **##** p < 0.01 vs. control. **C**. Effects of apoO silencing were measured using Western blots. β-actin served as a loading control. These experiments were performed three times, and the results of the densitometric analysis and one representative image are shown. ** p < 0.01 vs. NC; **##** p < 0.01 vs. control.

Three days after infection, HepG2 cells were visualized using fluorescence microscopy. A comparison between the bright-field and fluorescent images showed that most cells had green fluorescent signals when MOI = 20 (Figure 4A). The levels of apoO mRNA and protein were analyzed 5 days and 7 days after transfection, respectively. The level of apoO mRNA in cells transfected with apoO-specific-RNAi lentivirus was decreased by approximately 78% (P < 0.01) compared to the control cells (Figure 4B). ApoO protein was also down-regulated by approximately 80% in cells with the apoO-specific RNAi (P < 0.01; Figure 4C).

### Differentially expressed genes in apoO-silenced HepG2 cells

**Figure 5:**
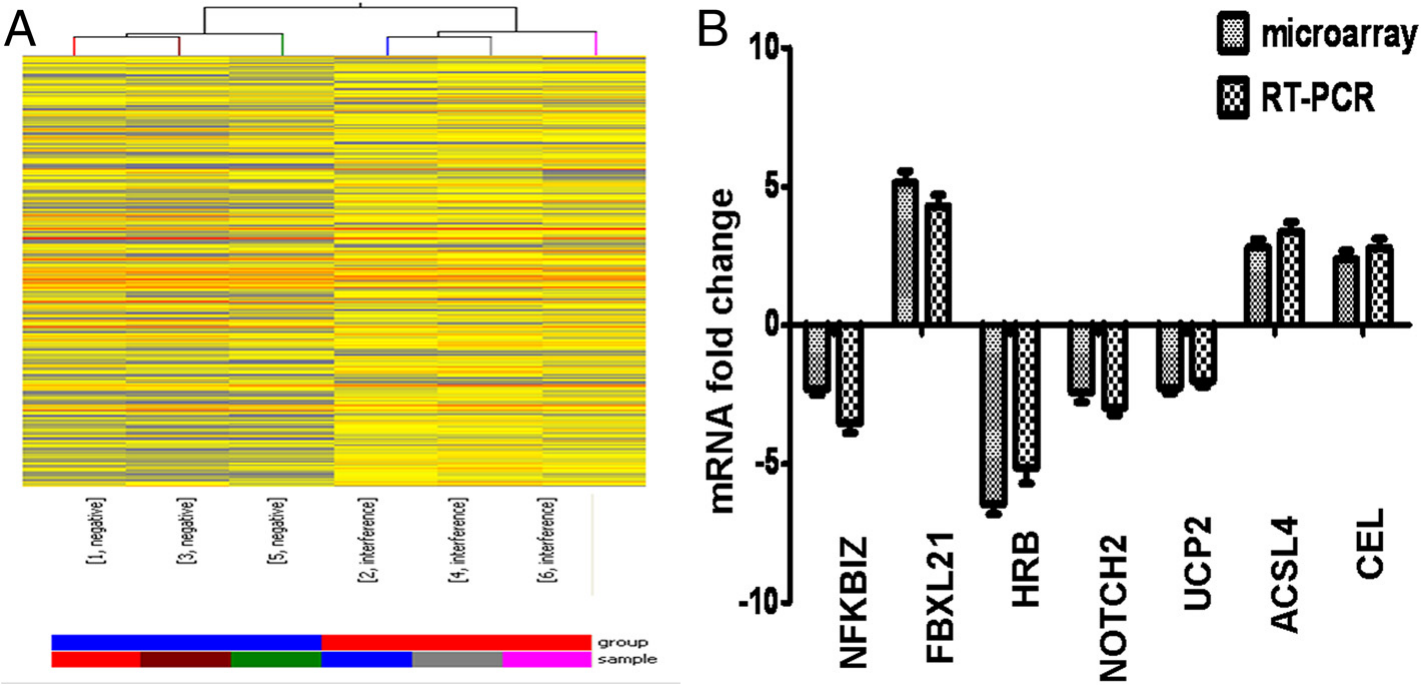
**Differentially expressed genes and verification. A**. Hierarchical clustering of differentially expressed genes in the negative control group vs. the interference group. The rows show individual genes, while the columns show individual tissue samples. Red denotes high expression and blue denotes low expression. **B**. Comparison of the expression levels of genes as fold-changes between the negative control group and the apoO-silenced group by microarray analysis and qRT-PCR. Assays were performed from each RNA sample in triplicate. Data were normalized using GAPDH as an endogenous control for RNA input. Fold-changes for these microRNAs from the microarray and qRT-PCR are shown as means ± S.E.M. (n = 6 for each group).

Comparison of mRNA levels between negative control cells (NC) and apoO-silenced cells (LV) revealed that many genes were differentially expressed. In summary, the expression of 282 genes was significantly altered: 192 genes were up-regulated and 90 genes were down-regulated (≥2 fold up- or down-regulated; Figure 5A, n = 6, P < 0.05). The expression of the *FBXL21* gene, which showed the strongest up-regulation, was increased by 5.142-fold, whereas the expression of the *HRB* gene, which showed the strongest down-regulation, was decreased by 6.485-fold. Of the genes with altered expression patterns, 18 were involved in lipid metabolism (Table 1) and 16 were involved in inflammation (Table 2). Moreover, gene ontology (GO) analysis identified involvement of the differentially expressed genes in several cellular biological processes, such as 1) glycerolipid metabolism, 2) glycerophospholipid metabolism, 3) sphingolipid catabolism, 4) membrane lipid catabolism, 5) cellular lipid metabolism, 6) phospholipid metabolism, 7) phosphatidylinositol metabolism, 8) the Notch signaling pathway, 9) steroid catabolism, 10) phospholipid biosynthetic processes, 11) glycerophospholipid biosynthetic processes, 12) sphingolipid metabolism, 13) response to superoxide, and 14) response to oxygen radicals (Table 3).

**Table 1:** Differential expressed genes related to lipid metabolism

**Gene name**	**NCBIGene ID**	**Fold change**	**FDR**	**P value**	**Description**
CEL	BC042510	2.383↑	0.999	0.030	Carboxyl ester lipase(bile salt-stimulated lipase)
CROT	BC051874	2.271↓	0.999	0.037	Carnitine O-octanoyltransferase
AGPAT2	NM_006412	2.025↓	0.999	0.015	1-acylglycerol-3-phosphate O-acyltransferase 2 (lysophospha-tidic acid acyltransferase, beta)
CYP4F11	NM_021187	2.490↓	0.999	0.014	Cytochrome P450, family 4, subfamily F, polypeptide 11
PI4KII	NM_018425	2.140↓	0.999	0.046	Phosphatidylinositol 4-kinase type II
PIP5KL1	NM_173492	2.171↓	0.999	0.017	Phosphatidylinositol-4-phosphate 5-kinase-like 1
G6PC2	BC104778	2.091↑	0.999	0.014	Glucose-6-phosphatase,catalytic,2
SERINC5	BC101281	2.586↑	0.999	0.018	Serine incorporator 5
SMPDL3B	NM_001009568	2.000↑	0.999	0.037	Sphingomyelin phosphodiesterase, acid-like 3B
ACSL4	NM_004458	2.803↑	0.999	0.017	Acyl-CoA synthetase long-chain family member 4
RBP1	NM_002899	2.031↑	0.999	0.044	Retinol binding protein 1, cellular
LASS1	NM_198207	2.018↑	0.999	0.014	LAG1 longevity assurance homolog 1 (S. cerevisiae)
HSD17B6	NM_003725	2.051↓	0.999	0.033	Hydroxysteroid(17-beta) dehydrogenase 6
PIGW	BC033540	2.201↑	0.999	0.016	Phosphatidylinositol glycan, class W
RGS16	NM_002928	2.936↑	0.999	0.023	Regulator of G-protein signalling 16
KLF5	NM_001730	2.192↓	0.999	0.016	Kruppel-like factor 5 (intestinal)
UCP2	NM_003355	2.248↓	0.999	0.028	Uncoupling protein 2 (mitochondrial, proton carrier)
UCP3	NM_003356	2.420↓	0.999	0.006	Uncoupling protein 3 (mitochondrial, proton carrier)

*Abbreviations:FDR* False discovery rate, ↑= increased, ↓= decreased.

**Table 2:** Differential expressed genes related to inflammation

**Gene name**	**NCBIGene ID**	**Fold change**	**FDR**	**P value**	**Description**
APH-1B	AB189172	2.372↓	0.999	0.040	Anterior pharynx defective 1 homolog B (C. elegans)
NFAT5	AJ243299	2.249↑	0.999	0.040	Nuclear factor of activated T-cells 5, tonicity-responsive
SLC37A2	AK074207	2.936↑	0.999	0.023	Solute carrier family 37 (glycerol-3-phosphate transporter), member 2
IL17A	BC066251	2.097↑	0.999	0.004	Interleukin 17 (cytotoxic T-lymphocyte-associated serine esterase 8)
LILRB2	NM_005874	2.014↑	0.999	0.046	Leukocyte immunoglobulin-like receptor, subfamily B (with TM and ITIM domains), member 2
RASGRF2	NM_006909	2.091↑	0.999	0.048	Ras protein-specific guanine nucleotide-releasing factor 2
NFKBIZ	NM_001005474	2.302↓	0.999	0.012	Nuclear factor of kappa light polypeptide gene enhancer in B-cells inhibitor, zeta
CD160	NM_007053	2.401↑	0.999	0.015	CD160 molecule
N2N	NM_203458	2.144↑	0.999	0.005	Notch homolog 2 (Drosophila) N-terminal like
NOTCH2	BC071562	2.082↓	0.999	0.008	Notch homolog 2 (Drosophila)
TNFSF15	BC074941	2.022↑	0.999	0.045	Tumor necrosis factor (ligand) superfamily, member 15
CCL23	NM_005064	2.570↑	0.999	0.017	Chemokine (C-C motif) ligand 23
DTL	BC033540	2.356↑	0.999	0.021	Denticleless homolog (Drosophila)
USP2	NM_171997	2.088↑	0.999	0.019	Ubiquitin specific peptidase 2
UCP2	NM_003355	2.248↓	0.999	0.028	Uncoupling protein 2 (mitochondrial, proton carrier)
UCP3	NM_003356	2.420↓	0.999	0.006	Uncoupling protein 3 (mitochondrial, proton carrier)

*Abbreviations:FDR* False discovery rate, ↑= increased, ↓= decreased.

**Table 3:** GO analysis of the differentially expressed genes

**Gene ontology**	**Fold enrichment**	**P value**	**Genes**
Glycerolipid metabolic process	3.350	0.0092	CEL//AGPAT2//SERINC5//PI4KII//PIGW//PIP5KL1
Glycerophospholipid metabolic process	3.833	0.010	AGPAT2//SERINC5//PI4KII//PIGW//PIP5KL1
Sphingolipid catabolic process	10.644	0.0148	SMPDL3B//CEL
Membrane lipid catabolic process	10.052	0.0165	SMPDL3B//CEL
Cellular lipid metabolic process	1.963	0.0198	ACSL4//CROT//UCP3//CEL//AGPAT2//SERINC5//PI4KII//SMPDL3B//PIGW//RBP1//PIP5KL1//LASS1
Phospholipid metabolic process	2.827	0.0199	AGPAT2//SERINC5//PI4KII//SMPDL3B//PIGW//PIP5KL1
Phosphatidylinositol metabolic process	2.674	0.0202	PI4KII//PIP5KL1
Notch signaling pathway	4.679	0.0263	NOTCH2NL//NOTCH2//APH1B
Steroid catabolic process	7.867	0.0264	CEL//HSD17B6
Phospholipid biosynthetic process	3.513	0.0273	AGPAT2//PI4KII//PIGW//SERINC5
Glycerophospholipid biosynthetic process	3.991	0.0394	AGPAT2//PI4KII//PIGW
Sphingolipid metabolic process	3.667	0.0486	SMPDL3B//LASS1//CEL
Response to superoxide	18.095	0.0052	UCP2//UCP3
Response to oxygen radical	16.45	0.0063	UCP2//UCP3

### Confirmation of microarray results by qRT-PCR

To validate the microarray results, we assessed the expression of a subset of genes with qRT-PCR, including: *NFKBIZ, FBXL21, HRB, NOTCH2, UCP2, ACSL4 and CEL*. The high concordance of expression levels found between the microarray analysis and the qRT-PCR results for these genes confirmed that the microarray data were reliable (Figure 5B).

## Discussion

The liver is the most metabolically active organ in the human body and is responsible for many vital functions, including lipid metabolism. Triglycerides (TGs) accumulate within hepatocytic lipid droplets. Notably, both the lipolysis and synthesis of TG can produce FAs and specific toxic lipid intermediates that activate intracellular inflammatory pathways. FA oxidation provides a major source of reactive oxygen species (ROS). During the process of hydrolysis, TG expands the availability of FA to metabolic pathways, such as peroxidation; increases ROS; and, subsequently, increases levels of oxidative stress. Oxidative stress results in the activation of several key pro-inflammatory signaling pathways, including the nuclear factor-kappa B (NF-κB) pathway [4]. Conversely, although TG synthesis decreases cellular FA concentrations, this process also generates other potentially toxic lipid intermediates [5]. Therefore, dysfunctional FA metabolism influences the cellular inflammatory state and is involved in the pathogenesis of liver disorders, such as non-alcoholic fatty liver disease (NAFLD) [6].

The present study demonstrates that the exposure of HepG2 cells to OA as well as TNF-α can result in the increased expression of apoO. To account for this phenomenon, we examined gene expression in apoO-silenced HepG2 cells with the use of microarrays. According to our microarray data, silencing apoO in HepG2 cells leads to the differential expression of several important lipid signaling and inflammation genes.

### FA metabolism

In comparison with the negative control cells, *ACSL4* was up-regulated in apoO-silenced HepG2 cells. *ACSL4* encodes an isoform of the long-chain acyl-CoA synthetase (ACSL), which catalyzes acyl-CoA synthesis by converting long-chain FA to acyl-CoA. African-American NAFLD patients over-express ACSL4 [7]; ACSL4 mRNA levels have been positively associated with liver TG concentrations [8]. Thus, increased *ACSL4* expression could indicate an up-regulation of TG synthesis.

Mitochondria are the major site of FA oxidation. *RGS16* encodes a protein which inhibits G protein-coupled receptor (GPCR)-stimulated FA oxidation in liver mitochondria [9]. Its expression increased after transfection. *CROT* and *CYP4F11* encode two key FA oxidation enzymes, respectively. In the peroxisome, storage of medium chain acyls slows down peroxisomal beta oxidation. When CROT activity increases, the level of medium chain acyls decreases as they are converted into acyl-carnitines [10]. In the microsome, another FA oxidation site, CYP4F11 is the predominant catalyst of FA omega hydroxylation [11]. Within this context, silencing of apoO with altered *RGS16*, *CROT* and *CYP4F11* expression would modulate not only FA oxidation rates but also cellular TG content.

### Inflammatory responses

The NF-κB protein family includes transcription factors that regulate crucial cellular processes, such as the inflammation response. *NFKBIZ* encodes a novel member of the IκB family, IκB zeta. IκB zeta associates with both the p65 and p50 subunits of NF-κB and inhibits the transcriptional activity and DNA binding of NF-κB [12]. USP2 is a ubiquitin-specific protease which is required for the phosphorylation of IκB and functions as an additional positive regulator of TNF-α-induced NF-κB signaling [13]. The protein encoded by *TNFSF15* gene is a cytokine that belongs to the tumor necrosis factor ligand family and is capable of activating NF-κB [14]. In addition, the two cytokines IL-17 and CCL23 may induce inflammatory gene expression by interaction with the NF-κB pathway [15,16]. As apoO silencing in HepG2 cells resulted in down-regulation of *NFKBIZ* and up-regulation of the pro-inflammatory molecules mentioned above, it is possible that apoO may exert anti-inflammatory effects through suppressing NF-κB pathway.

Notch signaling is involved in the inflammatory response [17]. There is complex crosstalk between the Notch 2 and NF-κB pathways as both pathways can exert either synergistic or antagonistic effects depending on different cellular contexts [18-20]. APH-1 is one of the four components of γ-secretase complex, which is responsible for the release of the notch intracellular domain (NICD) into the cytoplasm. These subunits are sufficient and required for γ-secretase activity [21]. The product of *N2N* gene is homologous to Notch 2. *In vitro*, N2N repressed the transcriptional activity of the Notch 2 protein in a dose-dependent manner [22]. Our microarray data showed that activation of NF-κB pathway was accompanied by impaired expression of *Notch2*, *APH-1B* and enhanced expression of *N2N,* suggesting the antagonistic effect between the NF-κB and Notch 2 signaling pathways in apoO-silenced HepG2 cells.

### Uncoupling proteins

Uncoupling protein (UCP) 2 and UCP3 are members of a mitochondrial carrier protein superfamily that controls the level of respiration coupling. The *UCP2* and *UCP3* genes are located together in a gene cluster, but the pattern of their expression is very different. UCP3 is primarily expressed in skeletal muscle tissue, whereas UCP2 is expressed widely. Although these two uncoupling proteins are thought to have similar physiological functions [23], the changes of *UCP2* expression levels in liver cells may be more significant. UCP2 can dissipate the proton gradient across the mitochondrial inner membrane to prevent the proton-motive force from becoming excessive, thus limiting mitochondrial ROS production. It acts as a sensor for mitochondrial oxidative stress and protects against oxidative damage by controlling the production of ROS [24]. UCP2 is also implicated in fat oxidation and the regulation of fat content. It functions as a metabolic switch that is involved in the choice of substrate oxidized by mitochondria which promotes FA metabolism over glucose utilization [25]. It could be speculated that reduction of *UCP2* expression in apoO-silenced HepG2 cells would lead to mitochondrial dysfunction accompanied by elevated ROS production and oxidative stress in hepatocytes. Subsequently, mitochondria might regulate the generation of ROS by altering the activity levels of enzymes that can affect FA oxidation. Therefore, reduction of UCP2 provides a possible mechanism in which FA metabolism and ROS-induced inflammatory responses can be simultaneously modulated in HepG2 cells after the silencing of apoO.

### Limitations

Some limitations of this study should be considered. Firstly, although the HepG2 hepatoma cell line is frequently used to study apolipoprotein metabolism, they are not the gold standard model for the study of the native liver. Therefore, gene expression changes related to apoO in our study may not reflect those that occur in liver *in vivo*. Secondly, we have only reported the result of an experiment that illustrated that apoO expression was dramatically affected by OA and TNF-α, but changes in inflammation and lipid metabolism genes in apoO-silenced HepG2 cells pretreated either with OA or TNF-α were not explored. Hence, the exact mechanisms involved in apoO-dependent changes in gene expression have yet to be elucidated. In addition, apoO, besides being secreted, could reside within cells where it is expressed [1]. It may affect hepatocytes as a paracrine and/or autocrine factor, or even as an intracellular protein. This study did not differentiate whether changes of gene expression are due to the secreted apoO, or due to the intracellular apoO.

## Conclusions

Our findings provide significant data to propose a role for apoO in HepG2 cells. Using a whole-genome microarray analysis, we have demonstrated that apoO may play an active role in FA metabolism in HepG2 cells by inhibiting TG synthesis and promoting FA oxidation. Furthermore, it is possible that apoO could suppress hepatic inflammation *via* the NF-κB and Notch 2 signaling pathways. UCP2 provides an underlying connection between the changes in expression levels of lipid metabolism and inflammatory response genes in apoO-silenced HepG2 cells.

## Materials and methods

### Cell culture

HepG2 cells and HeLa cells (originally obtained from the American Type Culture Collection, ATCC) were maintained in Dulbecco’s modified Eagle’s medium (DMEM; Invitrogen, Carlsbad, CA, USA) at 37°C under 5% CO_2_, supplemented with 10% fetal bovine serum (FBS; Invitrogen, Carlsbad, CA, USA).

For experimental incubations, cells were washed once in serum-free medium for 2 h. The medium was then replaced or cells were incubated with this medium with the addition of 1 mmol/L OA (Sigma, St. Louis, MO, USA) or 100 ng/mL TNF-α (Sigma, St. Louis, MO, USA) for 24 h.

### RNA isolation and qRT-PCR analysis

Total cellular RNA was isolated using Trizol reagent (Invitrogen, Carlsbad, CA, USA) according to the manufacturer’s instructions, and was quantified using a NanoDrop Spectrophotometer (Nanodrop, Rockland, DE, USA). cDNA was synthesized using a cDNA Synthesis Kit (Fermentas, Burlington, Ontario, Canada). qRT-PCR was performed with the SYBR Green Master Mix reagent (ABI, Foster City, CA, USA) in an ABI 7300 RT-PCR apparatus according to the manufacturer’s protocol. For each gene, qRT-PCR was run on each sample in triplicate. Transcript levels were normalized using GAPDH RNA quantification. The results of the qRT-PCR were statistically analyzed with SigmaStat software (SPSS). PCR primers used in the validation and stimulation tests are listed in Table 4.

**Table 4:** Real-time PCR primers used in the validation and stimulation tests

**Primer**	**Sequence**
NFKBIZ	F: 5′- GTTGTCTGATGGACCTGC −3′
	R: 5′- CTGTTTGGGTTCATTGAG −3′
FBXL21	F: 5′- TAAGTCCACTCATCCTGATCT −3′
	R: 5′- CAAAATGAGACTCCGACACA −3′
HRB	F: 5′- CAAAGAGGACAATCCCAGAG- 3′
	R: 5′- GATCAGGTCCAGGGTTGC −3′
NOTCH2	F: 5′- CCCAATGGGCAAGAAGTCTA −3′
	R: 5′- CACAATGTGGTGGTGGGATA −3′
UCP2	F: 5′- CGGTTACAGATCCAAGGA −3′
	R: 5′- ACCAGCCCATTGTAGAGG −3′
ACSL4	F: 5′- TTGCCATCTTCTGTGAGA −3′
	R: 5′- GGTAATCAGATAGGAAGCC −3′
CEL	F: 5′- TATGATCTGGATCTATGGAGG −3′
	R: 5′- CGACACGGTAGTTGAAGGT −3′
APOO	F: 5′- GGTGTCAGGAAACGTACTCCC −3′
	R: 5′- AACCCCCATTGAACCAAACTT −3′
GAPDH	F: 5′- GGAAGGTGAAGGTCGGAGTC −3′
	R: 5′- GCTCCTGGAAGATGGTGATGG −3′

### Protein extractions and Western blots

Extraction of cellular protein and Western blot analyses were carried out as previously described [26]. The protein bands were detected using ECL detection reagents (Pierce Biotechnology, Rockford, IL, USA) and quantified by densitometric analysis.

### Construction of the lentiviral vectors

Complementary DNAs containing both the sense and antisense oligo DNAs of the targeting sequences were synthesized, and were annealed into double-stranded DNA. The DNA products obtained were cloned into the lentiviral pFU-GW-iRNA vector (GeneChem, Shanghai, China) to construct the experimental vectors, which were then identified by PCR and DNA sequencing. The successfully constructed vectors were packaged using a packaging plasmid mix. The virus titers were tested. HeLa cells were infected by the lentivirus constructs, and the efficiencies of apoO interference were identified by qRT-PCR and Western blot.

### SiRNA silencing

The day before transfection, cells were seeded into 6-well culture plates at a density of 10^5^ cells per well. On the day of transfection, they were infected with the lentiviral constructs (either the apoO-silencing vector or the negative control vector) at a favorable MOI. At 12 h after transfection, the medium was replaced. Cells were collected for RNA isolation and qRT-PCR analysis 5 days after transfection. Seven days after transfection, cellular proteins were extracted and Western blot analyses were conducted.

### Microarray analysis

About 5 μg total RNA from each sample was used for labeling and array hybridization as follows: 1) reverse transcription was carried out using the Invitrogen Superscript ds-cDNA Synthesis Kit (Invitrogen, Carlsbad, CA, USA); 2) ds-cDNA was labelled using the NimbleGen one-color DNA Labeling Kit (Roche NimbleGen, Madison, WI, USA); 3) array hybridization was performed using the NimbleGen Hybridization System (Roche NimbleGen, Madison, WI, USA), followed by washing with the NimbleGen Wash Buffer Kit (Roche NimbleGen, Madison, WI, USA); 4) array scanning was carried out using the Axon GenePix 4000B Microarray Scanner (Molecular Devices Corporation). The data files were imported into Agilent GeneSpring Software (Agilent, version 11.5) for analysis. The gene expression levels in cells transfected with the efficient siRNA were normalized to that in cells transfected with the negative control siRNA. The microarray experiment was independently repeated in triplicate. Genes that were differentially expressed were identified by filtering the mean ratios of fold-changes from replicates using P-value thresholds (fold change ≥ 2 and P < 0.05) based on a *t*-test analysis. To determine the potential biological functions and pathways of differentially expressed genes, GO and pathway analyses were applied. Finally, Hierarchical Clustering was performed to show distinguishable gene expression profiling among samples.

### Statistical analysis

Data were depicted as means ± S.E.M. Statistical analyses were performed using SPSS software (Version 16.0, SPSS Inc., Chicago, IL, USA). Single comparisons were performed using the unpaired Student’s *t*-test with a value of p ≤ 0.05 considered as significant.

## Abbreviations

ApoO: Apolipoprotein O; hsCRP: High-sensitive C-reactive protein; OA: Oleic acid; TNF-α: Tumor necrosis factor-α; qRT-PCR: Quantitative real-time PCR; GO: Gene ontology; TG: Triglyceride; ROS: Reactive oxygen species; NF-κB: Nuclear factor-kappa B; NAFLD: Non-alcoholic fatty liver disease; ACSL: Long-chain acyl-CoA synthetase; GPCR: G protein-coupled receptor; NICD: Notch intracellular domain; UCP: Uncoupling protein; MOI: Multiplicity of infection.

## Competing interests

The authors declare that they have no competing interests.

## Authors’ contributions

CLW performed all experiments and statistical analysis and drafted the manuscript. BLY and SPZ conceived and designed this study, participated in discussion of the results and helped to revise the manuscript. All authors read and approved the final manuscript.
